# Determination of the Monoclonal Antibody Tocilizumab by a Validated Micellar Electrokinetic Chromatography Method

**DOI:** 10.1007/s10337-022-04148-w

**Published:** 2022-04-01

**Authors:** Sahar Zayed, Fathalla Belal

**Affiliations:** 1 Unit of Drug Analysis, Faculty of Pharmacy, Mansoura University, Mansoura, Egypt; 2 Pharmacy Department, Emergency Hospital, Mansoura University, Mansoura, Egypt; 3 Pharmaceutical Analytical Chemistry Department, Faculty of Pharmacy, Mansoura University, Mansoura, Egypt

**Keywords:** Tocilizumab, Micellar electrokinetic chromatography, Validation

## Abstract

Tocilizumab is a monoclonal antibody used in the treatment of several inflammatory and autoimmune diseases as well as cancers. Tocilizumab improves clinical outcomes and reduce mortality rates in patients with COVID-19 disease. A novel, simple and reliable method was developed to determine tocilizumab using micellar electrokinetic chromatography (MEKC). Separation of tocilizumab and the internal standard, methotrexate, was achieved with a background electrolyte consisting of phosphoric acid buffer and sodium dodecyl sulfate (SDS) with UV detection at 195 nm. The method was linear in the concentration range from 10 to 250 µg/mL with correlation coefficient greater than 0.995. The method was successfully applied to the analysis of human and rat plasma samples with good recoveries. Sample preparation involved protein precipitation followed by dilution of the supernatant. The intra‐ and inter-day precisions were less than 5%, the accuracy varied from − 2.71 to 3.84%. The proposed method has acceptable analytical performance and could be applied in future clinical and pharmacokinetic studies including anticancer therapy.

**Supplementary Information:**

The online version contains supplementary material available at 10.1007/s10337-022-04148-w.

## Introduction

Monoclonal antibodies are the fastest growing class of new drugs exhibit therapeutic benefits against various diseases [1, 2]. Tocilizumab (TCZ) is a monoclonal antibody that acts as an interleukin (IL)-6 receptor antagonist. TCZ plays an important role in the treatment of several inflammatory and autoimmune diseases as well as cancers [3–6]. Recent studies supported the use of TCZ in the treatment of critical or severe coronavirus disease 2019 (COVID-19) patients [7, 8].

Therefore, establishing reliable bioanalytical methods is essential in the preclinical and clinical evaluation of TCZ.

The literature survey reveals that, enzyme-linked immunosorbent assay (ELISA) [9–15] and electro-chemiluminescence assay (ECLA) [16] methods have been reported for the assay of TCZ in biological samples. Other analytical methodologies including size-exclusion chromatography ((SEC) HPLC–DAD) [17], cation-exchange chromatography ((CEX) UHPLC-DAD) [17], HPLC-FL [18] and HPLC–MS [19] have already been suggested for TCZ quantification. However, these methods are expensive, time-consuming and involve complicated chemical reactions, and not ideal for use in routine basis. Although HPLC is a rapid technique enabling highly efficient separations, it produce larger amount of waste products. It is commonly known that minimal sample preparation steps reduce the possibility of unreliable and inconsistent results. Thus, high efforts are made to reduce the number of experiments to shorten the time, while still maintaining low costs when handling complex biological samples.

Capillary electrophoresis (CE) is an important technique for analysing many pharmaceutical and biopharmaceutical substances. CE characterized by its simplicity, efficiency, short analysis time and little consumption of samples and reagents [20]. Micellar electrokinetic chromatography (MEKC) is one of the most widely used CE modes. It is a hybrid of electrophoresis and chromatography that is used for the separation of neutral solutes as well as charged ones [21]. MEKC proved to be a useful technique for various applications including bioanalytical separations [22–25]. Although there are a few CE studies on TCZ quantification/characterization [26–28], MEKC has never been optimized and validated for the quantification of TCZ in biological fluids.

The use of MEKC in basic buffers with positive potential was usually considered as the standard features in most of the MEKC works. In these conditions, the electroosmotic flow (EOF) is dominant and that drives the common anionic SDS micelles towards the cathode end. Under acidic pH (pH < 2.5; in the current work), the EOF is very weak or negligible, where the electrophoretic velocity of the micelles is the dominant motive force in the capillary. The micelles with the partially included analytes migrate to the anode and this characteristic MEKC is termed reversed migration MEKC (RM-MEKC) [29].

The aim of the present work was to develop and validate an analytical method that quantifies TCZ using MEKC technique with UV detection. Methodology described in this paper opens up the possibility of analyzing TCZ in human and rat plasma using simple protein precipitation that makes the method faster and easier. The developed method could be applied for further research to assess, for instance, the pharmacokinetics of TCZ.

## Materials and Methods

### Instrumentation

Experiments were performed with an Agilent 7100 Capillary Electrophoresis system (Agilent Technologies, Waldbronn, Germany) equipped with a diode-array detector. Data were recorded with Agilent Open LAB CDS software. Uncoated Fused-silica capillary 75 µm i.d × 50 cm (41.5 cm effective length) from Polymicro Technologies (Phoenix, AZ, USA) was used. A Metrohm® pH meter (Herisau, Switzerland) was used for pH measurement. A Model 800 centrifuge (Jiangsu Zhenji Instruments Co., Ltd., Jiangsu, China) were used for plasma preparation.

### Chemicals and Reagents

Actemra® (Roche, Basel, Switzerland) is a product of Chugai Pharmaceutical Co., Ltd. (Tokyo, Japan) was used as a representative TCZ reference material. All of the standard solutions of TCZ were prepared from this product. The product is supplied as a sterile, preservative-free protein solution at a concentration of 20 mg/mL for Intravenous Infusion. Methotrextare (MTX) as internal standard (IS) was obtained from Sigma Chemicals. Acetonitrile, methanol, 85% phosphoric acid were purchased from Sigma-Aldrich (Germany). Sodium dodecyl sulfate (SDS) and sodium hydroxide (Fisher Scientific, UK) were used. A 0.45 mm membrane filters (used to filter all sample and buffer solutions before CE analysis) were purchased from Phenomenex (Torrance, California, USA). Ultrapure water was obtained from a Milli-Q water purification system (Millipore, Bedford, MA, USA). Drug-free human plasma was from the blood bank of Mansoura University Hospital, Mansoura, Egypt. Drug-free rat plasma was purchased from Nile Center of Experimental Research (NCER), Mansoura, Egypt. The pooled plasma blanks were stored at − 20 °C prior to use.

### CE Conditions

Before the first use, the capillary was conditioned by flushing with 1.0 M NaOH for 60 min, then with de-ionized water for 30 min, and BGE for 30 min. At the beginning of each working day, the capillary was rinsed with 0.1 M NaOH for 10 min, de-ionized water for 5 min, and then with BGE for 10 min. Before each injection, the capillary was preconditioned with BGE (3 min) to maintain proper repeatability of run-to-run injections. Sample injections were performed hydrodynamically at a pressure of 50 mbar for 30 s and the capillary temperature was set at 20 °C. The voltage applied in the separation was − 15 kV and the wavelength of the UV detector was maintained at 195 nm. The BGE solution was prepared as follow: stock solutions of 200 mM phosphoric acid and 200 mM SDS were prepared, then mixed together in a ratio that produces final BGE containing 30 mM phosphoric acid (pH 2.1) and 60 mM SDS. The running buffer was passed through a 0.45 µm syringe filter and degassed by sonication for 5 min before use.

### Preparation of Standard Solutions

A stock standard solution of 2 mg/mL TCZ was prepared daily by diluting Actemra® in de-ionized water. MTX (IS) stock solution at a concentration of 400 µg/mL was prepared in methanol and de-ionized water (1:1, v/v). The stock solution of TCZ was further diluted with de-ionized water to get standard solutions ranging between 10 and 200 µg/mL at constant MTX (IS) concentration of 2.5 μg/mL. The stock solutions were stored at 4 °C and found stable for up to 72 h.

### Calibrations Standards and Quality Control Samples

Human plasma samples were spiked with the desired amount of TCZ stock solution, according to the required concentration. Standards for the calibration curve were prepared with the final concentrations of 10, 25, 50, 100, 150 and 250 μg/mL at constant MTX (IS) concentration of 2.5 μg/mL. The quality control (QC) samples were 25, 100 and 200 μg/mL as lower quality control (HQC), medium quality control (MQC) and higher quality control (LQC), respectively. A cross-matrix validation was performed to ensure that the rat plasma was comparable with the human plasma matrix. TCZ was spiked into rat plasma to make three quality controls (25, 100 and 200 μg/mL). Quality controls in rat plasma were used to evaluate the precision and accuracy against the human plasma standard curve with a range from 10 to 250 μg/mL.

### Sample Pretreatment Procedure

Each aliquot (100 µL) of human or rat plasma samples was added with 10 µL of MTX (IS) working solution and then with 200 µL of a mixture of acetonitrile and methanol (1:1, v/v). Afterwards, the mixture was vortex mixed for 30 s and centrifuged at 3000*g* for 10 min. Then, 100 μL of the supernatant was diluted with 300 μL de-ionized water. The contents were then transferred to the CE instrument for analysis. A blank experiment was performed simultaneously.

## Results and Discussion

### Method Development and Optimization

The possibility of better sensitive detection of TCZ under acidic conditions was investigated. To obtain an acceptable separation, effects of pH, buffer concentration, SDS concentration, separation temperature, applied voltage and injection time were studied. The parameters concerning MEKC method optimization are given in Supplementary Table 1.

**Table 1:** Analytical parameters for determination of the TCZ by proposed method

Parameter	Standard solution	Human plasma
Repeatability RSD (*n* = 5)		
Migration time (%)	1.35	1.89
Peak area (%)	2.12	2.48
Concentration range (μg/mL)	10-250	10-250
Regression equation	*y* = 0.1012x + 0.0317	*y* = 0.2153x−0.0785
Correlation coefficient (*R*^2^)	0.9998	0.9989
Detection limit (μg/mL)	2.85	3.12
Quantification limit (μg/mL)	9.36	9.74

#### Effect of Buffer pH

Phosphate buffer (phosphoric acid) was used at pH values between 1.8 and 3.8, the initial buffer concentration was set at 30 mM. To modify the pH of the buffer, NaOH solution was added to the BGE. As buffer pH increased, the EOF was gradually increased, which resulted in longer migration time and peak broadening. The best separation was achieved at pH 2.1 within a reasonable analysis time, so this was selected as the optimum pH value.

#### Effect of Buffer Ionic Strength

Phosphate buffer was investigated over the range from 20 to 60 mM. The peak resolutions and migration times slightly change at various buffer concentrations. However, the peak areas of TCZ increased with the increase in the concentration from 20 to 30 mM. At concentrations above 30 mM, the peak areas did not increase anymore and the effect of joule heat became more pronounced (≈ 72 µA). Therefore, 30 mM phosphate buffer was chosen for the following experiments.

#### Effect of SDS Concentration

The influence of SDS concentration was investigated from 20 to 80 mM during method development. At lower SDS concentrations (20–40 mM), TCZ showed a very poor peak shape and symmetry. By increasing SDS concentrations, better peak shape and better resolution were found. Nevertheless, SDS concentration greater than 60 mM resulted in increased method analysis time with more joule heating. Therefore, 60 mM SDS concentration was selected as the best compromise between peak shape and generated current.

#### Effect of Separation Voltage

The applied voltages in the range from − 10 to − 18 kV were tested. As expected, an increase in the voltage, led to a decrease in the migration times with increased peak efficiencies. Due to the big inner diameter (75 µm) of the capillary, applying higher voltages (− 18 kV) resulted in current disruption due to Joule heating. On the other hand, lower voltages at (− 10 kV) resulted in long migration time, along with broad peaks. Thus, the optimal voltage was set at − 15 kV which ensured short migration time, good resolution and acceptable current generated (≈ 55 µA).

#### Effect of Capillary Temperature

The influence of the capillary temperature on separation efficiency was studied from 15 to 25 °C.

Generally, when the temperature increases, the resistance of the buffer goes down and the generated electric current increases. As expected, an increase of the capillary temperature caused a decrease of migration times due to lower electrolyte viscosity. Capillary temperature at 25 °C resulted in an increase in peak asymmetry and reduced efficiency which might be due to the lower electrolyte viscosity and excessive Joule heating. Thus, the selected temperature was 20 °C as it provides the best compromise between peak shape and time of analysis.

#### Effect of Injection Time

To improve the sensitivity of the method, different injection times were tested from 10 to 40 s at 50 mbar. As the sample injection time increased, a marked increase of peak intensity was observed. However, higher injection times led to distorted peaks and decreased resolution. Therefore, 30 s was chosen as the optimum injection time.

#### Selection of the Detection Wavelength and the Internal Standard

The optimal wavelength was established experimentally with the aid of the photodiode array detector. The best sensitivity was achieved at 195 nm, thus, it was used for detection. Furthermore, no interference was encountered from the components from plasma matrix. The use of an internal standard is recommended to compensate injection errors and minor fluctuations of the migration time improving the quantitative analysis. Several drugs were tested including methotrexate (MTX) which was found to be a suitable candidate with well-resolved peak.

**Fig. 1:**
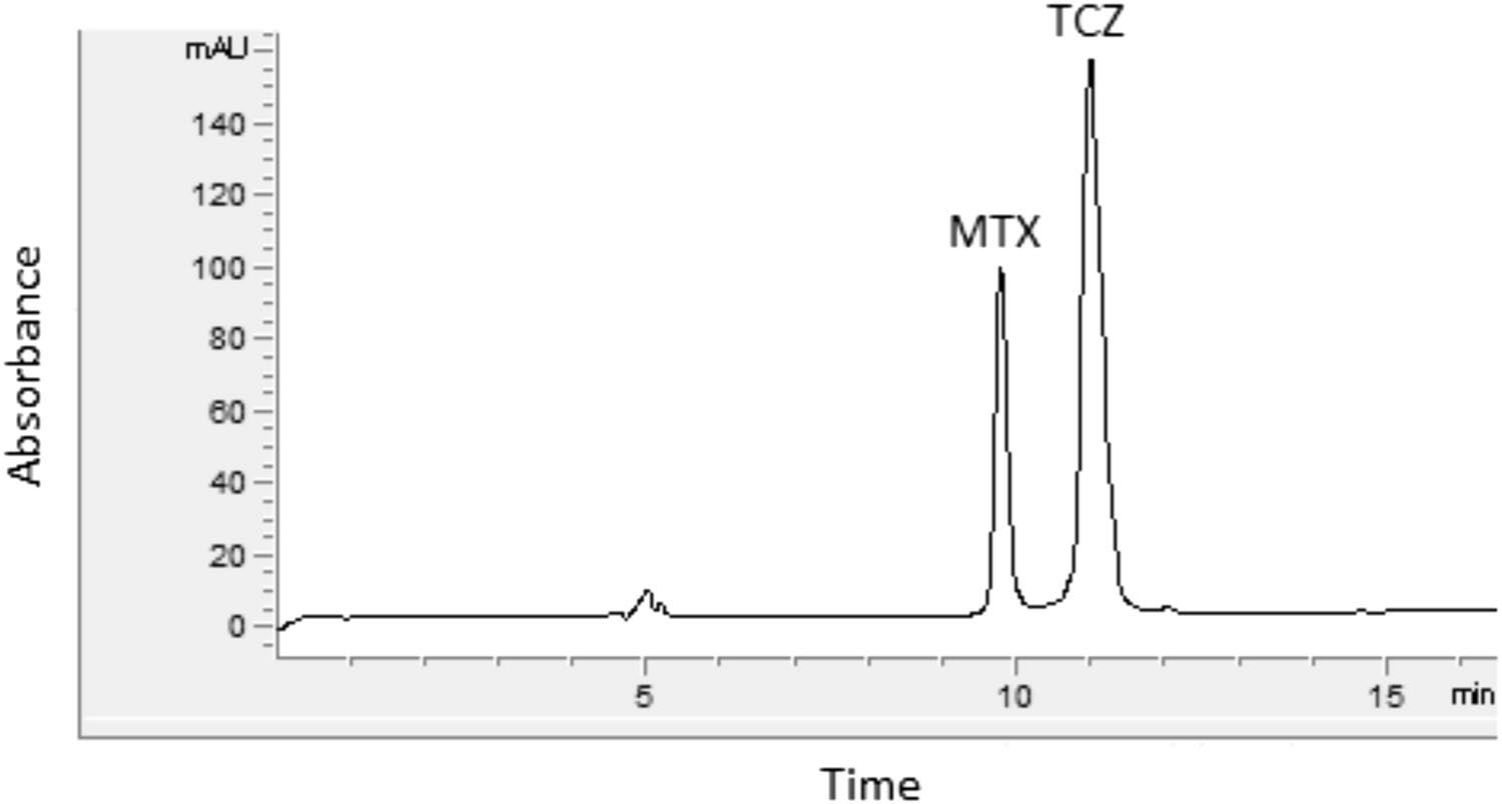
Typical electropherogram of standard solution of TCZ (150 μg/mL) and MTX (IS, 2.5 μg/mL) obtained under optimized analysis conditions. BGE composed by 30 mM phosphoric acid at pH 2.1 and 60 mM SDS; − 15 kV; 20 °C

A typical electropherogram of standard solution obtained under the optimized analytical conditions is shown in Fig. 1. Total analysis time was less than 15 min with a resolution factor of 3.69 (between TCZ and MTX).

### Sample Pretreatment

Sample matrices can dramatically influence resolution and detectability when directly injected into the CE instrument. Thus, a simple, economical and efficient sample preparation procedure is urgently needed. Protein precipitation was firstly investigated to attain cleaner samples with minimal endogenous interferences, but with considerable recoveries. The following solvents were tested: methanol, isopropanol, acetonitrile and a mixture of acetonitrile and methanol (1:1, v/v). Protein precipitation was obtained by adding 200 μL of each solvent/mixture to 100 μL of spiked plasma with the same vortexing and centrifugation times. The results showed that a mixture of acetonitrile and methanol (1:1, v/v) was the most efficient in extracting TCZ from plasma. To ensure good peak shape for TCZ and to reduce the matrix effect, the supernatant was diluted with water before the CE injection. For this purpose, several dilution factors were evaluated, including 1:1, 1:2, 1:3, 1:4 and 1:5. After employing a dilution factor of 1:3 (supernatant-water [v/v]), the peak of TCZ was symmetric and the matrix peaks nearly disappeared. So, a dilution factor of 1:3 was used in our formal assay validation.

### Method Validation

To demonstrate the suitability of the developed method, validation was carried out according to the guidelines set by the Food and Drug Administration (FDA) [30] for biological sample analysis. The validation parameters studied are:

#### Linearity, Detection Limits, Quantification Limits and Repeatability

The linearity of the method was evaluated by plotting calibration curves with six concentrations of TCZ in the 10–250 μg/mL range. The linearity regression equations were *y* = 0.1012x + 0.0317 (*r*^2^ = 0.9998); and *y* = 0.2153x-0.0785 (*r*^2^ = 0.9989) for standard and plasma, respectively; where x represents the concentration of TCZ and y represents the relative peak area (the corrected peak area of TCZ divided by the corrected peak area of internal standard (MTX). The corrected peak area is defined as the peak area divided by the migration time. The limit of detection (LOD) and the limit of quantification (LOQ) values were estimated based on a signal-to-noise ratio of 3 and 10 respectively. The LOD and LOQ values for TCZ are shown in Table 1. Repeatability of migration times and peak areas are introduced as suitability parameters. The obtained RSDs indicating reasonable repeatability of the proposed method (Table 1). It is noteworthy that the calibration range was broad enough to include the concentrations that have been reported in clinical trials and in real studies (9, 11, 12, 15, 16). The maximum plasma concentrations (*C*_max_) of TCZ was 88 ± 41 µg/mL after an intravenous dose of 4 mg/kg every 4 weeks; increasing up to 183 ± 86 µg/mL as the dose augmented to 8 mg/kg [9]. The estimated plasma concentrations of TCZ, in trough steady state, were 42 ± 27.4 µg /mL [11] and 62.1 ± 30.5 µg /mL [12] after a weekly subcutaneous administered dose of 162 mg. In addition, the average C_max_ after subcutaneous and intravenous administration of 162 mg TCZ, were 80.5–114 ± 5.89–26.4 µg/mL and 246 ± 24 µg/mL, respectively [15]. On the other hand, in Novartis Trial ELIANA, the C_max_ of TCZ measured by ECLA ranged from 99.5 to 161 µg/mL [16]. Therefore, the proposed method is suitable and has adequate sensitivity for determining TCZ concentrations in plasma.

#### Selectivity

**Fig. 2:**
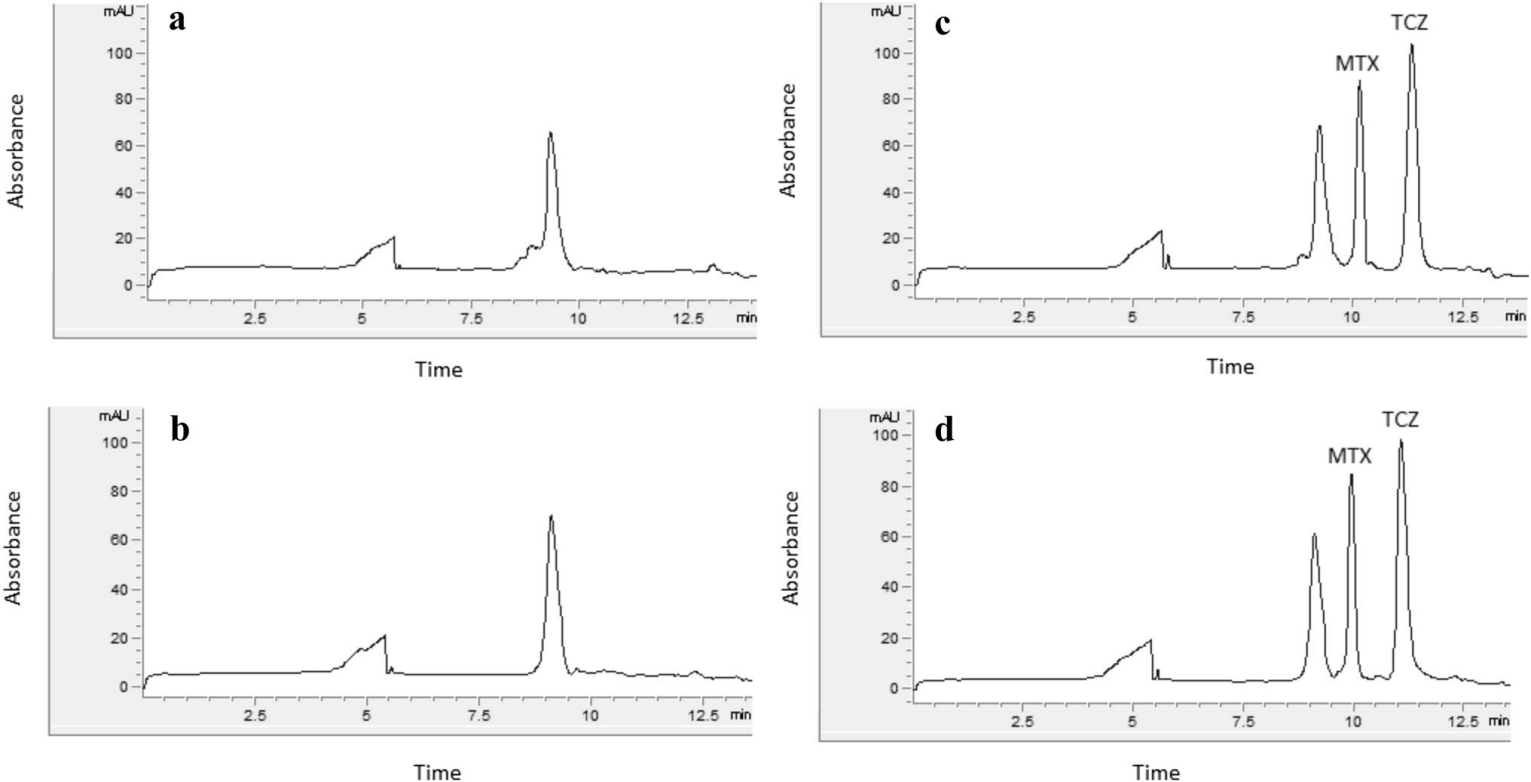
Typical electropherograms of (**a**) blank human plasma (**b**) blank rat plasma (**c**) spiked human plasma and (**d**) spiked rat plasma; TCZ (100 μg/mL) and MTX (IS, 2.5 μg/mL)

Selectivity of the method was verified by comparing the electropherograms of spiked and drug free plasma samples. For this purpose, spiked samples of TCZ (100 μg/mL) with MTX (2.5 μg/mL) and six randomly selected blank human and rat plasma samples were prepared and analyzed. No interfering peak was observed at the retention times of TCZ or the internal standard MTX (Fig. 2).

The method is therefore selective with no interference from endogenous plasma components.

#### Precision, Accuracy and Recovery

The precision and accuracy of the method were determined by assaying QC samples (*n* = 5) prepared in human and rat plasma with three concentration levels in the same day (intra-day) and over five days (inter-day). The degree of precision and accuracy is expressed as relative standard deviation (RSD%) and relative error (RE%), respectively. Table 2 shows satisfactory results in all QC samples. Intra-day percision was lower than or equal to 4.11%, while inter-day precision was lower than or equal to 4.58%. Intra-day accuracy ranged from—1.37 to 3.84% and inter-day accuracy ranged from—2.71 to 2.32%. The recovery of TCZ was determined by comparing the peak areas of QC samples with those obtained from corresponding standard solutions. As shown in Table 2, recoveries between 95.43 and 98.17% were obtained in all cases.

**Table 2:** Precision, accuracy and recovery data of TCZ in human and rat plasma

Concentration (µg/mL)	Intra-day			Inter-day			Plasma recovery (% ) Mean ± SD
	Mean (µg/mL)	Precision^a^ (RSD %)	Accuracy^b^ (RE %)	Mean (µg/mL)	Precision^a^ (RSD %)	Accuracy^b^ (RE %)	
Human plasma							
25 (LQC)	24.11	3.82	− 0.89	25.79	3.21	0.79	95.56 ± 3.78
100 (MQC)	101.41	3.25	1.41	97.29	4.58	− 2.71	96.38 ± 3.56
200 (HQC)	203.84	2.49	3.84	201.68	2.19	1.68	98.17 ± 2.91
Rat plasma							
25 (LQC)	26.55	2.65	1.55	23.71	2.77	− 1.29	96.77 ± 3.17
100 (MQC)	98.63	4.11	− 1.37	102.32	1.86	2.32	97.85 ± 4.22
200 (HQC)	201.67	2.33	1.67	198.87	3.72	− 1.13	95.43 ± 2.64

^a^Expressed as % RSD = (S.D./mean) × 100.

^b^Calculated as [(found conc. − actual conc.)/actual conc.] × 100.

#### Robustness

The robustness indicates the ability of the proposed method to resist minor changes in analytical conditions during routine analysis. Buffer concentration (30 mM ± 2 mM) and pH (2.1 ± 0.1), SDS concentration (60 mM ± 2 mM), injection time (30 ± 1 s), applied separation voltage (15 ± 1 kV) and capillary temperature (20 °C ± 1 °C) were the modified parameters to evaluate the robustness of the method. Analyses were carried out in triplicate and only one parameter was changed in the experiments at a time. The RSD values for migration times for the evaluated parameters were in the range from 1.13 to 1.68%, while RSD values for resolution of TCZ and MTX were in the range from 1.49 to 1.87% which demonstrate the robustness of the developed method.

#### Stability

The stability of standard working solutions was examined and no changes were observed within 4 h at room temperature (~ 22 °C). Stability of TCZ in human plasma samples was observed at HQC and LQC levels. The samples were analyzed in the following conditions: after 2 h in room temperature (~ 22 °C); after 7 days frozen at – 20 °C and after three freeze/thaw cycles (− 20 °C). Additionally, the stability of TCZ in processed samples was assessed at 4 °C during 8 h and autosampler at 10 °C during 4 h. Analysis was performed in triplicate and the concentration of TCZ measured after storage was compared to the respective initial value. According to the results obtained, TCZ was stable in the different handling and storage conditions. Stability data are summarized in Table 3.

**Table 3:** Stability of TCZ in different storage conditions

Storage condition	Concentration (μg/mL)	Mean (μg/mL)	RE (%)	Recovery (%) Mean ± SD
4 °C, 8 h	25 (LQC)	24.77	− 0.92	99.08 ± 1.24
	200 (HQC)	197.89	− 1.06	98.95 ± 2.8
− 20 °C, 3 freeze/thaw cycles	25 (LQC)	24.12	− 3.52	96.48 ± 3.4
	200 (HQC)	195.22	− 2.39	97.61 ± 1.9
− 20 °C, 7 days	25 (LQC)	24.11	− 3.56	96.44 ± 2.3
	200 (HQC)	194.78	− 2.61	97.39 ± 1.8
Processed samples (R.T., 2 h)	25 (LQC)	24.86	− 0.56	99.44 ± 0.67
	200 (HQC)	200.32	0.16	100.16 ± 1.34
^a^ Processed samples (10 °C, 4 h)	25 (LQC)	25.34	1.36	101.36 ± 1.22
	200 (HQC)	198.45	− 0.78	99.23 ± 0.76

^a^The processed samples were stored in an autosampler.

## Conclusion

A reliable and efficient MEKC method was developed and validated for the quantification of the monoclonal antibody tocilizumab (TCZ) for the first time. The method has good selectivity, accuracy and repeatability within analysis time of 15 min. Simple and quick sample preparation procedure was built, allowing for high sample throughput with high recovery and minimal matrix effect. The method was successfully applied to the analysis of TCZ in spiked human and rat plasma samples. The linear range obtained adequately covered the therapeutic range TCZ in the real samples. The novel MEKC method is a promising tool for pharmacokinetic and clinical use.

## Supplementary Information

Below is the link to the electronic supplementary material.

Supplementary file1 (DOCX 17 KB)
