# Supplementary material for: Determination of the Monoclonal Antibody Tocilizumab by a Validated Micellar Electrokinetic Chromatography Method
Source: Chromatographia. 2022 Apr 1;85(5):481–8. doi: 10.1007/s10337-022-04148-w (PMC8972641; doi:10.1007/s10337-022-04148-w)
Supplement: Supplementary file 1 — Supplementary file1 (DOCX 17 KB) [file 10337_2022_4148_MOESM1_ESM.docx]

**Supplementary Table 1. Optimization of the electrophoretic conditions of the proposed MEKC method.**

| **Variable** | **Values** | **Theoretical plates** | **Migration time**  **(min)** | **Symmetry** |
| --- | --- | --- | --- | --- |
| **Buffer pH** | pH 1.8 | 22650 | 10.24 | 0.87 |
|  | pH 2.1 | 24787 | 11.21 | 0.88 |
|  | pH 2.8 | 20648 | 12.66 | 0.93 |
|  | pH 3.8 | 15292 | 14.98 | 1.14 |
| **Buffer concentration (mM)** | 20 | 20445 | 11.17 | 0.86 |
|  | 30 | 24787 | 11.21 | 0.88 |
|  | 40 | 24978 | 11.42 | 0.92 |
|  | 60 | 23413 | 11.51 | 0.94 |
| **SDS concentration (mM)** | 20 | 16436 | 9.78 | 0.79 |
|  | 40 | 19778 | 10.39 | 0.82 |
|  | 60 | 24787 | 11.21 | 0.88 |
|  | 80 | 26734 | 13.41 | 1.01 |
| **Voltage (kV)** | -10 | 20489 | 13.45 | 1.08 |
|  | -12 | 21175 | 12.64 | 1.02 |
|  | -15 | 24787 | 11.21 | 0.88 |
|  | -18 | 26315 | 10.13 | 0.92 |
| **Temperature (ºC )** | 15 | 22145 | 11.72 | 0.78 |
|  | 20 | 24787 | 11.21 | 0.88 |
|  | 25 | 15149 | 10.89 | 1.24 |
| **Time injection (s)** | 10 | 21982 | 11.23 | 0.89 |
|  | 20 | 23713 | 11.21 | 0.88 |
|  | 30 | 24787 | 11.21 | 0.88 |
|  | 40 | 22976 | 11.19 | 0.86 |
